# Task relevance determines binding of effect features in action planning

**DOI:** 10.3758/s13414-020-02123-x

**Published:** 2020-09-10

**Authors:** Viola Mocke, Lisa Weller, Christian Frings, Klaus Rothermund, Wilfried Kunde

**Affiliations:** 1 Department of Psychology, University of Würzburg, Würzburg, Germany; 2 Department of Psychology, University of Trier, Trier, Germany; 3 Department of Psychology, Friedrich-Schiller-University Jena, Jena, Germany

**Keywords:** Action planning, Motor control, Binding, Effect anticipations

## Abstract

Action planning can be construed as the temporary binding of features of perceptual action effects. While previous research demonstrated binding for task-relevant, body-related effect features, the role of task-irrelevant or environment-related effect features in action planning is less clear. Here, we studied whether task-relevance or body-relatedness determines feature binding in action planning. Participants planned an action A, but before executing it initiated an intermediate action B. Each action relied on a body-related effect feature (index vs. middle finger movement) and an environment-related effect feature (cursor movement towards vs. away from a reference object). In Experiments 1 and 2, both effects were task-relevant. Performance in action B suffered from partial feature overlap with action A compared to full feature repetition or alternation, which is in line with binding of both features while planning action A. Importantly, this cost disappeared when all features were available but only body-related features were task-relevant (Experiment 3). When only the environment-related effect of action A was known in advance, action B benefitted when it aimed at the same (vs. a different) environment-related effect (Experiment 4). Consequently, the present results support the idea that task relevance determines whether binding of body-related and environment-related effect features takes place while the pre-activation of environment-related features without binding them primes feature-overlapping actions.

## Introduction

How do humans plan motor actions? A possible, so-called ideo-motor, view on this process originates from the idea that we generate motor activities by setting up a mental representation of the perceptual effects that a certain motor activity will produce (Greenwald, 1970; James, 1981; Shin, Proctor, & Capaldi, 2010; Stock & Stock, 2004). Anticipating an action effect, which is “a change of sensory input that is triggered by a bodily movement” (Pfister, 2019, p. 154), should reactivate the bodily movement to which the action effect has been associated through previous experience. Indeed, there is now ample evidence that such perceptual representations mediate action production (Elsner & Hommel, 2001; Kunde, Koch, & Hoffmann, 2004; Pfister, 2019; Pfister & Kunde, 2013; Shin & Proctor, 2012). In other words, motor activities seem to be mentally represented and planned in terms of those perceptual events that a to-be-accessed motor activity will foreseeably produce.

### Feature codes in action planning

Perceptual events in general and perceptual effects that mediate action planning in particular are likely coded in terms of features (Frings, Hommel, et al., 2020; Frings, Koch, et al., 2020; Hommel, 1998, 2004, 2009; Hommel, Müsseler, Aschersleben, & Prinz, 2001). The idea that action planning is based on features is not new, as for example Rosenbaum (1980) argued that motor activities are prepared as motor programs with free slots that are filled by specific feature values during action planning or, respectively, the filling in of these feature values is in fact the planning process (see Leuthold & Jentzsch, 2011, for corresponding evidence). According to the theory of event coding (TEC), specifying a single feature is usually not sufficient to plan an action (Hommel et al., 2001). Rather, codes of multiple action effect features would become activated in a first step. At this point, the merely activated features should prime other action plans with overlapping features. As a second step, the activated feature codes would become integrated, or bound, through associative connections now resulting in interference instead of facilitation of partly overlapping action plans.

Importantly, the authors describe action plans as temporary composites of feature codes that describe to-be-produced external, or distal, events. That means, while they clearly rule out the possibility of event coding on the basis of proximal information – that is, neural codes or muscular innervation patterns – they argue that depending on the intended action effect feature codes can represent attributes of any kind of perceptual effect. Following this logic, it should be possible to plan actions by means of anticipated body-related feedback as well as feature codes of environment-related action effects that have an even more distal and oftentimes more artificial nature (Hommel et al., 2001). To clarify, planning a right index finger keypress might not only comprise representations of the anticipated tactile or proprioceptive impression of the right index finger movement (body-related action effects), but also of the sound (e.g., the click of the keyboard) or of some visual consequences that this movement might reliably produce (e.g., a certain letter on a computer screen, environment-related action effects). This basic underlying idea of action planning by means of to-be-produced effects should be kept in mind for the remainder of the present work. That is, because when using the term “feature” in the present work, we constantly refer to features of to-be-produced action effects, rather than features of the motor responses that produce these effects. Also, for the sake of brevity, we refer to features of body-related or environment-related effects as body-related and environment-related features.

Pfister (2019) argued that in many experimental tasks representing actions by body-related features (e.g., which finger to use to press a certain key) should be sufficient to achieve the task goal. Contrarily, using feature codes relating to environmental action effects might only be favorable if representing the action with feature codes of its body-related effects alone is in some way disadvantageous. For instance, this seems to hold true when the environmental effects are highly similar to the corresponding body movements (e.g., a cursor movement on screen mirroring a hand movement; Shin & Proctor, 2012, Experiment 2). In that case, environment-related features even become part of action representations when instructions demand participants to attend to body-related effects and ignore environment-related effects (see also Janczyk, Pfister, & Kunde, 2012, for related findings on hand-tool compatibility effects). Furthermore, when instructions explicitly demand participants produce environment-related events, actions are likely to be represented by environment-related features (Hommel, 1993). Such instructions can for example force participants to pay attention to changes in a display instead of their own movements, hence making the environment-related effects more salient (Janczyk et al., 2015, Experiment 2).

In particular, the latter aspect that increased attention on (or saliency of) environment-related effects promotes the integration of such effects in action representations leads to the question which role task-relevance of action effects plays in event coding (i.e., whether a feature relating to a particular action effect has to be part of the action plan for the actor to perform the correct action). According to the authors of TEC, all features on task-relevant effect dimensions should have a higher basic activation level than those on irrelevant effect dimensions due to a process called intentional weighting (see Memelink & Hommel, 2013). As a result, if planning an action activates such a task-relevant feature code, its activation level should be higher than that of activated irrelevant feature codes. A higher activation level of a feature might increase the chances of being bound to other features. Consequently, task-relevance might determine the integration of feature codes in action plans. In other words, the question remains whether a task-irrelevant feature is bound less likely in the action plan than a relevant feature, if it is bound at all.

### Partial feature overlap costs

As mentioned above, the unique idea of the feature approach pursued here is that features are temporarily bound together. Such binding should have consequences for other actions that occur in close temporal proximity to the planning process. Firstly, binding a feature while planning a certain action might render this feature less accessible for other actions, which require this feature as well. A second, but surely not incompatible, possibility is that this feature is still accessible for other actions but reactivates other, unwanted features to which it is still bound (Frings, Hommel, et al., 2020a; Frings, Koch, et al., 2020b; see *General discussion* section for a more thorough discussion of the different mechanisms).

To illustrate such costs, consider a study by Stoet and Hommel (1999, Experiment 2). These authors asked participants to plan an index finger movement with the left or right hand (which likely involves binding of the features *left* or *right* and *hand*, action A). While participants were planning this movement, that is, before its eventual execution, a pedal action with the left or right foot was requested (which likely involves binding the features *left* or *right* and *foot*, action B). In line with the binding hypothesis, initiating the pedal action was delayed when it relied on a feature also used to concurrently plan the hand action, that is, in the partial feature-overlap condition (e.g., a *left foot* action while a *left hand* action was planned), as compared to a pedal action in the no feature-overlap condition (e.g., a *right* foot action while a *left* hand action was planned). Crucially, in the design by Stoet and Hommel (1999), both feature dimensions available to plan the actions (*left* versus *right* and *hand* versus *foot*) were body-related, as they referred to spatio-anatomical characteristics, and task-relevant.

Other work that adopted this design (Fournier, Behmer, & Stubblefield, 2014; Mattson & Fournier, 2008; Mattson, Fournier, & Behmer, 2012) also used the required hand (*left* or *right*) as potentially overlapping feature dimension (notably, again body-related and task-relevant). These studies also revealed performance costs for action B in the partial feature-overlap condition (i.e., when it required the same hand as a previously planned action A) compared to a no-overlap condition in which action B required the other hand (see also Fournier & Gallimore, 2013; Fournier, Gallimore, Feiszli, & Logan, 2014, for similar observations with movement direction as an overlapping feature dimension). Remarkably, such partial repetition costs even occur when both actions make use of different modalities, with action A being a manual response with the *right* or *left* hand and action B a vocal response that imposes a demand on working memory (uttering "right" or "left" as a response to a visual stimulus, Fournier et al., 2010, Experiments 1 and 3). Therefore, both action plans can overlap regarding the same task-relevant feature dimension although the features refer to entirely different body-related effects depending on the action (the experience of pressing a key vs. uttering a word).

### Partial feature overlap benefits

Interestingly, such costs of partial feature overlap as compared to no feature overlap have not always been obtained. For example, Kunde, Hoffmann, and Zellmann (2002, Experiment 3) asked participants to plan a *left-* or *right-*hand movement (task-relevant, body-related feature), which would foreseeably produce a *high* or *low* tone (task-irrelevant, environment-related feature) for action A. However, before executing this movement, participants had to execute Action B, a *weak* or *forceful* finger press (task-relevant, body-related feature) that equally foreseeably produced a *high* or *low* tone. Consequently, both actions could predictably either produce the same or different tones. This design resulted in a partial overlap condition (e.g., when participants planned a *right* hand movement, which would produce a *high* tone, and executed a *weak* keypress, which resulted in a *high* tone) and a no overlap condition (e.g., when participants instead executed a *weak* keypress, which resulted in a *low* tone). If binding took place in the same way as in the above-described experiments, a similar pattern of results, that is, feature overlap costs, should occur. Contrarily, the *weak* or *forceful* actions were initiated faster if they resulted in the same rather than a different tone to the planned (*left* or *right* hand) action. That is, participants’ performance was superior with partial feature overlap as compared to no feature overlap. Following TEC, this finding suggests that while features of the to-be-produced tones did affect performance in concurrently executed actions, these features were apparently not bound into an action plan, as they did not interfere with the executed actions. The authors argued that facilitation of actions that share features of a certain environment-related outcome (a tone in this case) with a currently planned action might be quite useful as this would allow quick replacement of an initially planned action with a functionally equivalent one if, for sudden reasons, the initially planned action cannot be carried out (which might thus be termed a “functional equivalence” benefit). This interpretation is in line with a study by Janczyk and Kunde (2014) in which participants first planned an index or middle finger keypress, which would foreseeably produce a certain action effect. In some trials, they were then asked for a freely chosen keypress with the middle or index finger of the other hand. Importantly, participants tended to overcome their preference for using the homologous finger when switching fingers produced the same action effect as planned (vs. a different one),

In fact, partial overlap benefits as observed by Kunde et al. (2002) seem to occur whenever feature activation, but not binding, takes place for action A or action B. Regarding the former, Stoet and Hommel (1999, Experiment 3) showed that a lack of time and incentive for planning action A in advance led to better performance in the partial overlap condition than the no overlap condition. The authors explained this pattern in the sense that feature codes were cued and hence pre-activated, but not yet bound when participants executed action B. Regarding lacking integration of action B features, Wiediger and Fournier (2008) and Fournier, Wiediger, and Taddese (2015) found partial overlap benefits when they had participants perform a visually guided reach action with the *right* or *left* hand (action B) while holding a *right-* or *left-*hand action in preparation (action A). According to the authors, visually guided reach actions carried out online (i.e., adjusted while moving according to the discrepancy between current and intended hand position) invoke automatic visuomotor mechanisms and thus, unlike actions that base on advance planning, do not interfere with concurrently held action plans (for reviews of motorvisual facilitation and impairment see Thomaschke, Hopkins, & Miall, 2012a, 2012b).

### Two possible explanations: Task-relevance versus body-relatedness

What are the reasons for observing partial feature overlap costs in some cases but partial feature overlap benefits in other cases despite sufficient planning of action A and cognitive control required for action B? Based on the previous literature review we see two not mutually exclusive explanations. First, whether features are integrated into an action plan might be a matter of feature relevance (*task-relevance hypothesis*). Specifically, only those features that are used to distinguish between action alternatives might be bound to form an action plan. It seems likely that features like *left* and *hand* are rather relevant to distinguish a left hand movement from a right foot movement as used by Stoet and Hommel (1999). By contrast, the tones that were produced by certain movements in the study by Kunde et al. (2002) were task-irrelevant. They were consistently produced by certain finger movements, but neither did the instructions emphasize these tones, nor did they ask to produce them. Participants might thus have relied on features other than those that relate to the produced tones to distinguish between action alternatives.

It should be noted that this hypothesis is not all trivial, as in many situations other than action planning there is clear evidence for the binding of even irrelevant features into event files, such as bindings of responses and task-irrelevant distractor features (Frings, Rothermund, & Wentura, 2007; Rothermund, Wentura, & De Houwer, 2005) or short-term binding of responses to response-evoked perceptual feedback (Dutzi & Hommel, 2009; Elsner & Hommel, 2001; Hommel, 2005, Experiment 2; Moeller, Pfister, Kunde, & Frings, 2019). However, the bindings in all of these studies resulted from actual action execution and not action planning (i.e., a top-down process resulting from the internally driven anticipation of action effects instead of a bottom-up process arising from the experience of an actual response-effect episode). So, the task relevance hypothesis affords empirical testing in the case of feature-based action planning.

Another possible explanation relates to the type of events that such features describe. The participants in the study by Stoet and Hommel (1999) produced by their movement nothing perceptible other than the observable body movement itself. Thus, the perceptual event that represents that movement likely included only features that relate to the body itself. By contrast, in the study by Kunde et al. (2002), the participants’ movements produced a tone, which, like other (e.g., visual) effects, has a body-external nature. Consequently, features like *high* and *low*, when relating to a tone, can code a body-external event. Perhaps only features that relate to body-related events, but less so features of body-external events, become bound when it comes to planning an action (*body-relatedness hypothesis*).

In a recent study, Moeller, Pfister, Kunde, and Frings (2019) studied stimulus-response-effect (S-R-E) episodes in their entirety. In their design, a task-relevant stimulus (e.g., a letter) was accompanied by an irrelevant distractor (e.g., a color) and prompted a certain response (i.e., a task-relevant body-related effect such as a left index finger movement), which in turn produced a certain task-irrelevant perceptual effect (e.g., a tone). The authors found that features of the irrelevant distractor (the color) were bound to features of the task-relevant body-related effect (the finger movement) but not of the task-irrelevant environment-related effect (the tone). Bindings between task-relevant (body-related) and task-irrelevant (environment-related) effect features (the finger movement and the tone) were also clearly demonstrated. The authors explain the fact that there was binding of irrelevant distractor features to body-related effect features, but no binding to environment-related effect features, with the task-relevance of the former and the irrelevance of the latter. However, one could equally well view this as preliminary evidence for different potentials of features of body-related and body-external effects to become bound. To sum up, it is still unclear whether task-relevance and/or body-relatedness of action effects determine feature binding in action planning.

### The present research

**Fig. 1:**
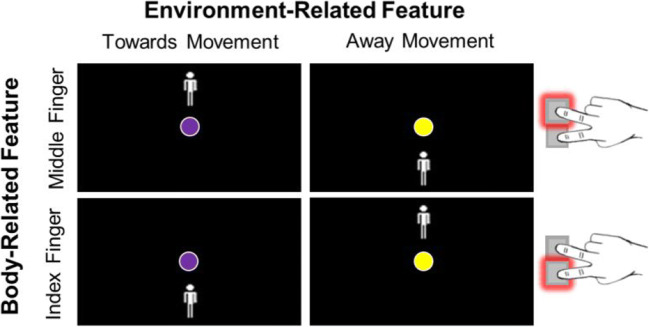
Basic paradigm adopted from Giesen and Rothermund (2016) with orthogonal manipulation of the task-relevant environment-related and the task-relevant body-related feature in action A. A purple cursor suggests a movement towards and a yellow cursor a movement away from the stick figure

The present research aims to clarify under which conditions binding of action effect features occurs during action planning. Specifically, we aim to test the task relevance hypothesis and the body-relatedness hypothesis described above. To do so, we asked participants to plan and carry out actions that comprised both a body-related feature and an environment-related feature (adopted from Giesen & Rothermund, 2016). Importantly, this paradigm allowed to orthogonally combine body- and environment-related features, and to render the environment-related effect features task-relevant or irrelevant. To illustrate this, consider Fig. 1. Participants were asked to move a round cursor on a computer screen either towards or away from a reference object (i.e., a stick figure). Thus, each of the participants’ correct responses to certain stimuli (i.e., pressing the correct of two response keys) produced one of two environment-related effects. These effects (i.e., cursor movements on screen) encompassed the features *towards* and *away*, respectively, and the color of the cursor indicated which kind of environment-related effect participants should produce. Depending on the position of the stick figure, this required a keypress with either the *index* or the *middle* finger. Please note, following ideo-motor theory, action plans rely on features of perceptual effects rather than muscular innervation patterns. From that perspective, the features *index* and *middle* are meant to describe the origin of the sensory changes that a motor pattern of a corresponding finger will bring about (i.e., the body-related action effects, such as the change in visual motion or tactile stimulation that comes with a corresponding finger movement), rather than the muscular activity, which, according to this theory, is mentally inaccessible.

Participants were asked to plan such an action (action A). However, before its eventual execution, that is, at varying time points after the announcement of the to-be-planned action A, another action (B) with varying degrees of feature overlap was requested for immediate execution. Specifically, the first initiated action B could share neither feature with the concurrently planned action A, or it could share one feature, or both (see Table 1). Please note, for the first time, this study allows for the full design including no, partial, and full-feature overlap, whereas previous research relied on the comparison of conditions with no and partial overlap alone (e.g., Fournier et al., 2015; Kunde et al., 2002; Stoet & Hommel, 1999).

**Table 1:** Example of effect features for action B and the resulting feature-overlap conditions while planning a middle finger keypress with a cursor movement towards the stick figure for action A (i.e., A: towards, middle)

Action B (while planning action A: towards, middle):		
	*Body-related feature*	
*Environment-related feature*	Same (middle) finger	Different (index) finger
Same (towards) movement	Full repetition (B: towards, middle)	Partial feature overlap (B: towards, index)
Different (away) movement	Partial feature overlap (B: away, middle)	Full alternation (B: away, index)

Unlike in most previous studies, we decided to present the stimulus display for action A not only during the initial planning phase but again when action A is actually to be executed. This could potentially reduce participants’ incentive for planning action A compared to a design without second stimulus presentation. However, in foreshadowing the results we show in various ways that planning action A actually occurred. More importantly, though, this design enabled us to prevent that participants simply memorized the finger to be used in action A (i.e., only the body-related feature would become part of the action plan). Specifically, to ensure that participants properly planned action A at the beginning of each trial (a prerequisite for influences of feature overlap on action B), participants had to detect catch-trials (10% of all trials) in which one of the to-be produced action effects at the end of the trial (i.e., either the finger that is to be used or whether the cursor will move towards or away from the stick figure) differed from the initial planning phase (see *Procedure* section for details). By doing so, participants had to include both features in their action plan. This way, we ensured equal task relevance of the body- and the environment-related action effect, which is crucial for disentangling the influences of body-relatedness and task relevance in binding. Also, introducing catch-trials enabled us to identify those participants who refrained from planning action A at all.

We further manipulated the time interval available for planning action A for exploratory purposes as well as to prevent participants from responding prematurely. Stoet and Hommel (1999) found significant binding effects when they let participants plan a more complex action for 3,350 ms. Hence, the time interval during which our participants could plan action A (1,500 vs. 2,000 ms, i.e., 1,000-ms presentation of the cue for action A and subsequently 500- or 1,000-ms interstimulus interval, ISI) should be sufficient for action planning in the current design.

Our analyses focused on performance in action B, which was emitted before any other efferent activity had occurred. Performance in action A was also assessed, but performance in this task is less easy to interpret, as it might be affected by feature overlap as well as by peripheral biomechanical factors from having just executed action B before (e.g., muscular priming or fatigue due to using the same finger twice in a row).

According to TEC, observing inferior performance in the first initiated action B, if it partly shares features with the concurrently planned action A as compared to a condition with full or no overlap, would suggest binding of these features in action planning. Previous work on bindings between stimulus and response features further suggests that performance in full-feature-overlap conditions is equivalent to no feature overlap (Hommel, 1998, 2004). Thus, in case that binding occurs, a characteristic interaction of repetition/alternation of the features of actions A and B is predicted. More specifically, we expect both reaction times (RTs) and error rates to be higher when either the body-related feature or the environment-related feature overlaps between action A and action B than when either both or none of the features overlap.

It should be noted that we took great care to avoid any sort of binding to certain stimulus characteristics that might otherwise occur. Specifically, a set of three different colors cued every towards (e.g., red, green, or purple) or away movement (e.g., blue, gray, or yellow), respectively. By doing so, every display in a trial (the cue for the planned action A, the stimulus for the first initiated action B, and the stimulus for the finally requested action A) contained a different cursor color so that no retrieval of features by stimulus color was possible.

With this basic paradigm, we conducted four experiments. Experiments 1 and 2 tested whether there is binding of features of environment- and body-related effects when both are equally task-relevant. In Experiment 3, we examined whether binding of features of environment-related effects occurs if they become task-irrelevant. Experiment 4 tested whether features of task-relevant environment-related events can be activated in advance even if they cannot be bound because of lacking body-related features that would be necessary to form a full-fletched action plan.

## Experiment 1

Experiment 1 aimed to test whether binding of features of environment-related action effects occurs, providing these features are equally task-relevant as features of body-related effects. If binding of features occurred, the initiation of action B should suffer when there is partial overlap with the features of the concurrently planned action A, as compared to full feature repetition or full feature alternation. On top of this interaction, there might be main influences of repetition of either body- or environment-related features. For example, responding for action B might be generally faster and/or more accurate if the body- or environment-related features overlap with the planned action A, respectively.

### Method

#### Participants

We conducted an a priori power analysis for the described binding effect, that is, the mean difference between the partial overlap conditions and the full alternation/repetition conditions for RTs of action B, by means of a two-tailed paired samples *t*-test using G*Power (Faul, Erdfelder, Lang, & Buchner, 2007). This yielded a minimum required sample size of *n* = 34 to detect a medium-sized effect (*d*_*z*_ = 0.50) with a power of 1–β = .80 and α = .05. Please note, the effect of feature overlap in related studies is typically larger (e.g., *d* = 1.01 in Stoet and Hommel, 1999), and thus our assumption of a medium-sized effect is rather conservative. We recruited a total of 34 participants via an online participant pool management platform of the University of Würzburg. The study was performed in accordance with the Declaration of Helsinki (Rickham, 1964) and had been approved by the local ethics committee (Ethikkommission des Institutes für Psychologie der Humanwissenschaftlichen Fakultät der Julius-Maximilians-Universität Würzburg, GZEK 2019-39). All participants gave their written informed consent before participation and received financial compensation of 10€. We excluded all participants who did not detect any catch-trials throughout the experiment (i.e., trials in which either the body- or the environment-related feature changed, *n* = 7) to ensure a sufficient degree of planning action A. Furthermore, we excluded one additional participant who failed to produce ten correct trials in one or more experimental cells. As a result, with the final sample size of *n* = 26 (17 females, *M*_*age*_ = 29.1 years, *range*_*age*_ = 20–55 years) the smallest possible effect size that could be detected with a power of 1–β = .80 and α = .05 was slightly higher than initially planned (i.e., *d*_*z*_ = 0.57).

#### Apparatus and stimuli

**Fig. 2:**
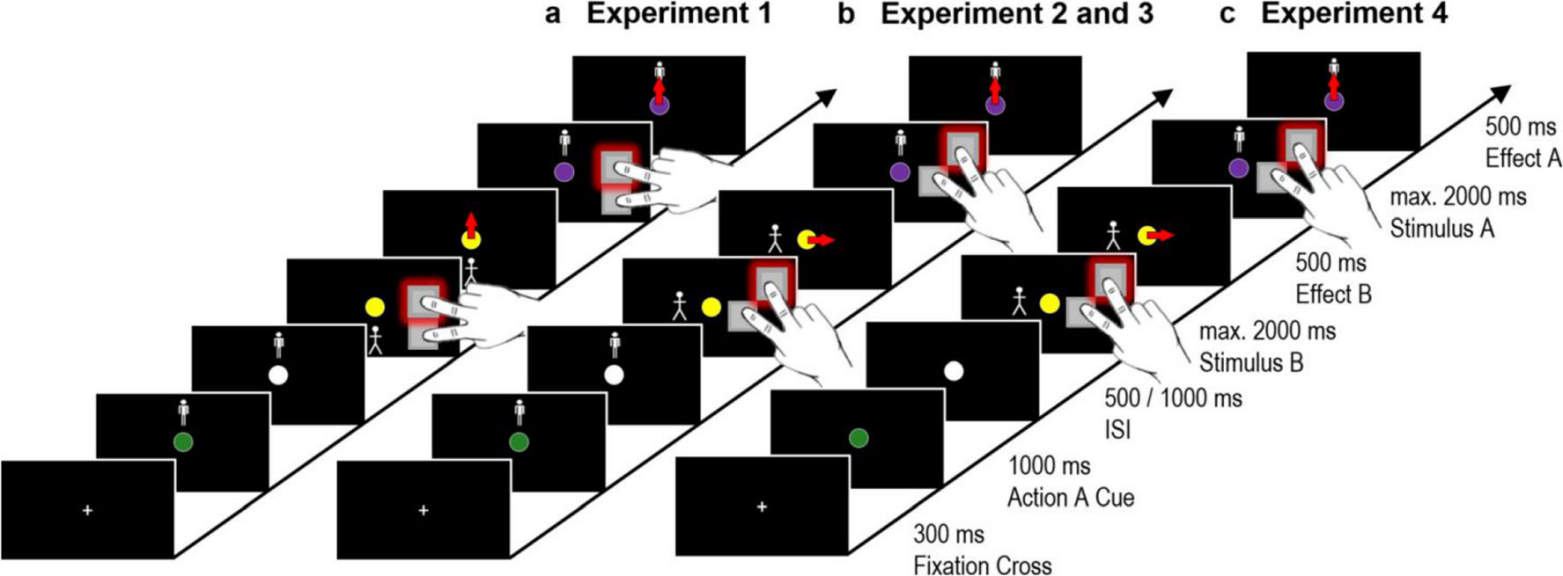
Exemplary partial feature overlap trial in Experiments 1 (**a**), 2 and 3 (**b**), and 4 (**c**) with a middle finger keypress producing a cursor movement towards the stick figure in action A and an away movement in action B. Response keys were the, slightly offset, K and M keys in Experiment 1 and external keys in Experiments 2–4. Note, the required actions are depicted here near the screen due to space limitations. They were carried out on a table below the screen in the actual experiment. Stimuli are not drawn to scale

Participants sat in front of an LCD monitor (24-in., BenQXL2411, BenQ) with a resolution of 1,920 × 1,080 pixels and a 100-Hz refresh rate. Stimuli were presented on screen using the E-Prime 2.0 software (Psychology Software Tools, 2002). Each display presented a round cursor in the center of the black screen that could move upwards or downwards (see Fig. 2). This resulted in a movement towards or away from a white stick figure, which could appear either on the top or bottom of the screen. To emphasize the distinctness of action A and action B, different stick figures were presented as reference objects for these two actions.

The cursor color signified which action participants had to plan and perform. For half of the participants, a red, green, and purple cursor indicated a towards movement, and a blue, gray, and yellow cursor indicated an away movement. For the other half of the participants, the mapping was reversed. The colored stimuli could trigger a keypress with the index or the middle finger, depending on the position of the stick figure (above or below the cursor).

Responses were given on a standard QWERTZ keyboard with the K key (pressed with the right middle finger) resulting in an upwards cursor movement and the M key (pressed with the right index finger) resulting in a downwards movement. Furthermore, participants responded to catch trials by pressing the D key.

#### Procedure

The experiment started with two practice instances, in which the participants were familiarized with the setup. Then, they worked through ten experimental blocks of 64 trials each with breaks after each block. Figure 2a illustrates one exemplary trial. Each trial started with a white fixation cross appearing for 300 ms, which was followed by display A (i.e., a colored round cursor in the middle of the screen and a stick figure above or below the cursor), during which participants should plan action A (e.g., a middle finger keypress that would produce a cursor movement towards the stick figure when seeing a stick figure above a green cursor). After 1,000 ms, the cursor turned white for a certain ISI (500 or 1,000 ms). Thereafter, display B appeared with another stick figure above or below a differently colored cursor. At this point, participants had 2,000 ms to respond to this display (e.g., with a middle finger keypress that made the cursor move away from the figure as a response to the figure being below a yellow cursor). After a correct keypress with the index or middle finger (i.e., the body-related effect) as instructed by the cursor color and dependent on the stick figure position, they observed the respective cursor movement towards or away from the stick figure for 500 ms (i.e., the environment-related effect). Subsequently, display A appeared again for a maximum of 2,000 ms and participants now had to execute the pre-planned action A. After pressing the correct key, they observed the respective cursor movement for 500 ms. Importantly, while the last display showed a stick figure and a colored cursor that asked for the pre-planned action A, the color of the cursor always changed with regard to the presented color at the beginning of the trial (e.g., as planned, a middle finger keypress that would produce a cursor movement towards the stick figure now as a result of seeing a stick figure above a purple cursor). This was achieved by having three colors that all signified the same towards-away movement. Thus, the stick figure appeared in the same position and the new cursor color entailed the same environment-related effect as in the beginning. Yet, in about 10% of the trials (catch-trials), display A suggested a different action than the pre-planned action A, because either the stick figure switched position as compared to the beginning of the trial or the requested environment-related effect changed (towards instead of away or vice versa) as suggested by the cursor color. Participants had to detect these catch-trials by responding with a separate key (D).

In case of an erroneous response (i.e., responding during the first display or ISI, pressing the wrong response key for action A or B, not responding in time for action A or B, missing a catch-trial or incorrectly indicating a catch-trial; see Table 2 for frequencies), an error message appeared for 1,000 ms and the trial terminated without later replacement. The subsequent trial started after an intertrial interval of 500 ms.

**Table 2:** Percentage of error trials within total trials for each experiment. Trials terminated as soon as an error occurred; therefore, errors are mutually exclusive. Ten percent of trials were catch-trials. Correct trials comprise correct non-catch-trials and correctly detected catch-trials. Values in a row might not add up to 100% due to rounding errors

Experiment	Premature responses	*Action B*		*Action A*				Correct trials
		Commission errors	Delayed responses	Commission errors	Delayed responses	Missed catch-trials	Incorrectly indicated catch-trials	
1	3.9	7.2	0.6	3.8	0.5	4.3	1.0	78.7
2	3.5	6.2	0.4	5.3	0.8	4.3	0.8	78.7
3	1.6	3.5	0.4	0.7	0.1	3.1	0.5	90.0
4	0.8	4.6	0.1	5.5	0.3	5.6	0.4	82.7

#### Design

The experiment followed a 2 × 2 × 2 repeated-measures design, with trial-wise manipulation of the three within-subject factors body-related feature overlap (same vs. different), environment-related feature overlap (same vs. different) and ISI (500 ms vs. 1,000 ms). Dependent variables were RTs and error rates for actions A and B.

#### Data analysis

The data and syntaxes for statistical analyses of all experiments, as well as the preregistrations for Experiments 3 and 4 adhere to the disclosure requirements and are publicly available on the Open Science Framework (https://osf.io/3xush/). The first two blocks served to familiarize participants with the task and were not further analyzed. Moreover, we excluded all trials in which participants responded prematurely from all analyses.

For the RT analysis of action B, we further only considered trials in which responses for action B as well as action A (to ensure sufficient planning of action A) were correct. For the action A analysis, we additionally excluded all correctly detected catch-trials. To account for outliers, we then excluded all trials with RTs deviating more than 2.5 standard deviations from the participant’s respective cell mean separately for both actions.

For the error-rate analysis of actions A and B, the respective RT outliers were discarded again and, for action B, all trials with erroneous responses for action A were excluded (again, to omit trials without sufficient planning). Error rates were then calculated as the percentage of relevant errors within the sum of erroneous and correct responses. For action B, relevant errors were commission errors (i.e., wrong keypresses) and delayed responses, and for action A additionally missed and incorrectly indicated catch-trials. Subsequently, we conducted repeated-measures analyses of variance (ANOVAs) on both RTs and error rates with the factors body-related feature overlap, environment-related feature overlap, and ISI.

For comparability with previous work that mainly reported this effect-size measure, we additionally present a Cohen *d*_*z*_ effect-size measure for the effect of main interest, that is, the RT difference between partial overlap conditions and full/no feature-overlap conditions in action B collapsed over both ISI conditions, obtained by a post hoc two-tailed paired-samples *t*-test. Lastly, for an additional check of whether participants planned action A in advance, we compared the RTs for action A and action B with a post hoc paired-samples *t*-test (see Table 4).

### Results

#### Action B

**Fig. 3:**
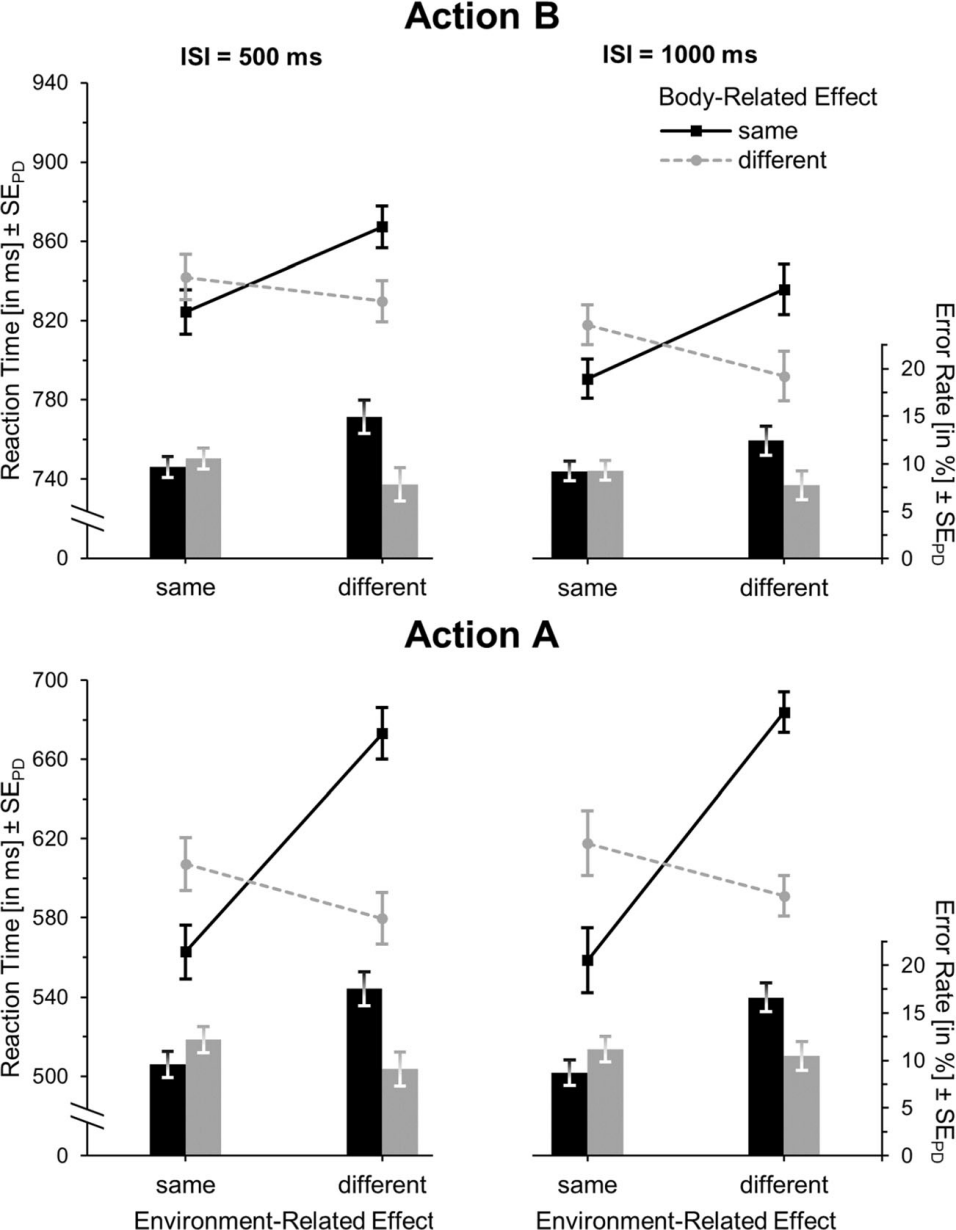
Mean reaction times (lines) and error rates (bars) for action B (upper panel) and action A (lower panel) in Experiment 1. Both features (full-repetition), no feature (full-alternation), or only one of the features (partial-overlap conditions) are identical in action A and action B. Within each environment-related feature-overlap condition, error bars represent standard errors of the paired differences (see Pfister & Janczyk, 2013) between the body-related feature-overlap conditions

Table 3 and the upper panel of Fig. 3 shows the results for action B. Regarding RTs, the main effects of environment-, *F*(1,25) = 2.79, *p* = .11, η_p_^2^ = .10, and of body-related feature overlap with action A, *F*(1,25) = 2.06, *p* = .16, η_p_^2^ = .08, failed to reach significance. However, responding was generally faster with a long compared to a short ISI, *F*(1,25) = 40.99, *p* < .001, η_p_^2^ = .62. Most importantly, there was a significant cross-over interaction between environment- and body-related feature overlap, *F*(1,25) = 17.38, *p* < .001, η_p_^2^ = .41. Specifically, reactions were slower in the partial feature-overlap conditions (averaged over both conditions: *M* = 841) than in the full-alternation or repetition conditions combined (*M* = 809, *t*(25) = 4.17, *p* < .001, *d*_*z*_ = .82). Furthermore, neither the environment-related, *F*(1,25) = 0.34, *p* = .57, η_p_^2^ = .01, nor the body-related feature overlap, *F*(1,25) = 0.05, *p* = .82, η_p_^2^ < .01, interacted significantly with ISI. Similarly, the three-way interaction did not reach significance, *F*(1,25) = 1.18, *p* = .29, η_p_^2^ = .05.

**Table 3:** Means (and standard errors of the means) of reaction times (RTs) and error rates according to interstimulus interval (ISI), and environment-related and body-related feature overlap for actions A and B for Experiment 1 (n = 26)

			ISI							
			500 ms				1,000 ms			
			Environment-related effect				Environment-related effect			
		Body- related effect	Same		Different		Same		Different	
Action B	RT	Same	824	(26)	867	(28)	791	(26)	836	(26)
		Different	842	(26)	830	(28)	818	(26)	792	(24)
	Error rate	Same	9.6	(1.8)	14.9	(2.8)	9.2	(1.6)	12.4	(2.4)
		Different	10.5	(1.9)	7.8	(1.7)	9.3	(1.9)	7.7	(1.8)
Action A	RT	Same	563	(35)	673	(43)	559	(36)	684	(44)
		Different	607	(40)	580	(36)	618	(44)	591	(42)
	Error rate	Same	9.6	(1.4)	17.6	(2.5)	8.7	(1.4)	16.6	(2.5)
		Different	12.2	(1.6)	9.1	(1.3)	11.2	(2.0)	10.5	(1.7)

The analysis of error rates failed to yield a significant main effect of environment-related feature overlap, *F*(1,25) = 3.60, *p* = .07, η_p_^2^ = .13, and ISI, *F*(1,25) = 2.20, *p* = .15, η_p_^2^ = .08. Conversely, responding was more accurate when action B relied on a different compared to the same finger than the planned action A, *F*(1,25) = 16.06, *p* < .001, η_p_^2^ = .39. Importantly, the interaction between environment- and body-related feature overlap was again significant, *F*(1,25) = 14.20, *p* = .001, η_p_^2^ = .36. Again, neither the interaction of ISI and environment-related feature overlap, *F*(1,25) = 0.09, *p* = .77, η_p_^2^ < .01, nor of ISI and body-related feature overlap, *F*(1,25) = 0.36, *p* = .55, η_p_^2^ = .01, nor the three-way interaction, *F*(1,25) = 2.42, *p* = .13, η_p_^2^ = .09, were significant.

#### Action A

The RTs of action A (see Fig. 3, lower panel) were significantly faster when both actions produced the same as opposed to different environment-related effects, *F*(1,25) = 38.10, *p* < .001, η_p_^2^ = .60, and with finger alternation compared to repetition, *F*(1,25) = 8.86, *p* < .01, η_p_^2^ = .26. It should be noted though that, as for action B, environment- and body-related feature overlap interacted in the sense that responses were slower in the partial compared to the full or no feature-overlap conditions, *F*(1,25) = 59.24, *p* < .001, η_p_^2^ = .70. The main effect of ISI was not significant, *F*(1,25) = 1.93, *p* = .18, η_p_^2^ = .07, and ISI did not significantly interact with environment-related feature overlap, *F*(1,25) = 1.42, *p* = .24, η_p_^2^ = .05, or body-related feature overlap, *F*(1,25) = 0.86, *p* = .36, η_p_^2^ = .03. There was also no three-way interaction, *F*(1,25) = 0.46, *p* = .51, η_p_^2^ = .02.

Error rates for action A were lower with repetition rather than alternation of the environment-related effect with action B, *F*(1,25) = 18.98, *p* < .001, η_p_^2^ = .43, and with alternation rather than repetition of the body-related effect, *F*(1,25) = 8.64, *p* < .01, η_p_^2^ = .26. Importantly, as in the RT analysis, the interaction between environment- and body-related feature overlap was again significant, *F*(1,25) = 28.95, *p* < .001, η_p_^2^ = .54. There was no significant main effect of ISI, *F*(1,25) = 0.17, *p* = .69, η_p_^2^ < .01, and neither ISI and environment-related feature overlap, *F*(1,25) = 1.05, *p* = .32, η_p_^2^ = .04, nor ISI and body-related feature overlap, *F*(1,25) = 0.92, *p* = .35, η_p_^2^ = .04, nor all factors, *F*(1,25) = 0.77, *p* = .39, η_p_^2^ = .03, interacted.

### Discussion

Experiment 1 revealed clear evidence for binding of features of body-related and environment-related effects in action planning. Initiating action B was facilitated if that action shared both or none of its features with a concurrently planned action A as compared to partial feature sharing. Importantly, in this experiment, both the environment- and body-related features were equally task-relevant. This clearly shows that even features that relate to environment-related effects become integrated into action plans, providing they are task-relevant, thereby supporting the task relevance hypothesis.

On top of this indication of feature binding, there was a tendency (action B) for a general benefit if both actions shared the same environment-related feature (significantly so for action A). This is preliminary support for the idea that an action that aims at the same environment-related effect as another prepared action generally benefits from the environment-related feature overlap with that action.

It should be noted that, although for both actions the task was the same, the RTs for action A were lower than those for action B (see Table 4). This strongly indicates that participants indeed planned action A in advance. Also, the data pattern did not heavily depend on the ISI, that is, the time interval between the offset of the first display and the request to initiate action B. Most importantly, the specific interaction pattern of body-related and -external feature overlap was already present 1,500 ms after announcement of action A (i.e., with an ISI of 500 ms). This suggests that binding of the relevant features of action A occurred rather quickly.

**Table 4:** Paired samples *t*-tests of the mean differences between action A and action B reaction times for each experiment. Cohen’s *d* calculated according to Dunlop, Cortina, Vaslow, and Burke (1996)

Experiment	Action A		Action B		*df*	*t*	*p*	Cohen’s *d*
	Mean	SD	Mean	SD				
1	606	198	823	128	25	6.42	< .001	1.24
2	695	260	835	124	37	3.44	.001	0.65
3	513	102	708	102	33	14.22	< .001	1.91
4	752	166	728	122	34	1.18	.245	0.16

Before considering these results in more detail, however, we have to deal with a possibly problematic aspect of the design: Whenever there was a partial feature repetition, the position of the stick figure on the screen that determined whether the prepared towards or away movement required an index or middle finger keypress did repeat (see Fig. 2a). For example, when a movement *towards* the stick figure with the *index* finger was prepared for action A, the stick figure was at the bottom of the screen. If now a *towards* movement with the *middle* finger was requested for action B, the stick figure changed its position to the top. However, when both features repeated or both changed, the stick figure position always remained the same. For example, when a movement *towards* the stick figure with the *index* finger was prepared for action A, the stick figure was at the bottom of the screen. If now an *away* movement with the *middle* finger was requested for action B, the stick figure remained at the bottom of the screen. In other words, the benefit for full feature repetition or alternation might be because the displays signaling the requested action contained fewer changes.

## Experiment 2

To consolidate the obtained results with a higher power as well as address the problem of varying changes of displays that signaled actions A and B, we conducted a second experiment and modified the setup in one respect. For action A, the towards or away movement of the cursor was always prepared in the vertical dimension, while the towards or away movement for action B was requested always in the horizontal dimension (see Fig. 2b). Consequently, the stick figures always changed their positions between displays used for preparing action A and requesting action B from a vertical to a horizontal position, independent of whether action B came with full, partial, or no feature repetition of action A.

### Method

#### Participants

To account for attrition in Experiment 2, we recruited a total of 47 participants. Seven participants failed to detect any catch-trials, one participant had less than ten correct trials in at least one experimental cell and one participant terminated the experiment during the first block. Consequently, we reached a power of 1–β = .85 for a *d*_*z*_ = 0.50 and α = .05 with a sample size of *n* = 38 (25 females, *M*_*age*_ = 27.2 years, *range*_*age*_ = 19–52 years).

#### Apparatus and stimuli

For action B, the stick figure appeared now on the left or right side of the cursor. Therefore, we now used diagonally aligned external keys so that an index finger keypress moved the cursor downwards for action A and leftwards for action B, while a middle finger keypress moved the cursor upwards for action A and rightwards for action B. Apart from these changes, the method was as in Experiment 1.

### Results

#### Action B

**Fig. 4:**
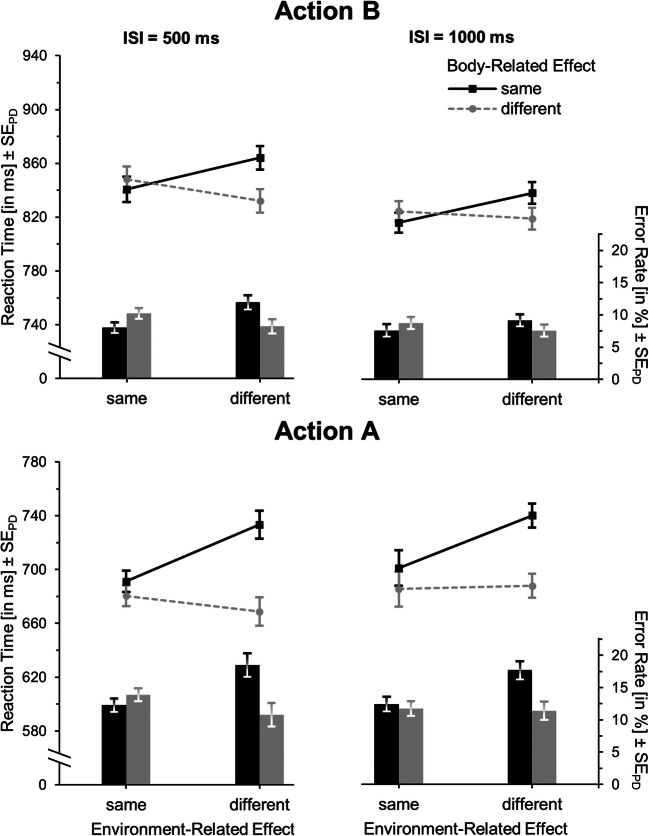
Mean reaction times (lines) and error rates (bars) for action B (upper panel) and action A (lower panel) in Experiment 2. Either both features (full repetition), no feature (full alternation), or only one of the features (partial overlap conditions) are identical in action A and action B. Within each environment-related feature-overlap condition, error bars represent standard errors of the paired differences (see Pfister & Janczyk, 2013) between the body-related feature-overlap conditions

Table 5 and the upper panel of Fig. 4 presents the results for action B. Again, the RT main effects of environment-related, *F*(1,37) = 0.79, *p* = .38, η_p_^2^ = .02, and body-related feature overlap, *F*(1,37) = 3.46, *p* = .07, η_p_^2^ = .09, failed to reach significance. Responses on trials with long ISIs, however, were faster than on short ISI trials, *F*(1,37) = 19.85, *p* < .001, η_p_^2^ = .35. Again, the interaction between environment- and body-related feature overlap was significant, *F*(1,37) = 13.72, *p* = .001, η_p_^2^ = .27, with longer RTs in the partial feature-overlap conditions (*M* = 844) than in the full alternation or repetition conditions (*M* = 827, *t*(37) = 3.70, *p* = .001, *d*_*z*_ = 0.60). As in Experiment 1, neither the environment-related, *F*(1,37) = 0.45, *p* = .51, η_p_^2^ = .01, nor the body-related feature overlap, *F*(1,37) = 0.72, *p* = .40, η_p_^2^ = .02, interacted significantly with ISI. Also, there was no significant three-way interaction, *F*(1,37) = 0.76, *p* = .39, η_p_^2^ = .02.

**Table 5:** Means (and standard errors of the means) of reaction times (RTs) and error rates according to ISI, and environment-related and body-related feature overlap for actions A and B for Experiment 2 (n = 38)

			ISI							
			500 ms				1,000 ms			
			Environment-related effect				Environment-related effect			
		Body- related effect	Same		Different		Same		Different	
Action B	RT	Same	841	(19)	864	(21)	817	(21)	839	(21)
		Different	848	(23)	832	(22)	825	(21)	820	(22)
	Error rate	Same	7.9	(1.7)	11.8	(1.9)	7.6	(1.7)	9.1	(1.6)
		Different	10.1	(1.9)	8.1	(1.7)	8.7	(1.8)	7.5	(1.7)
Action A	RT	Same	691	(44)	733	(45)	701	(45)	740	(44)
		Different	680	(42)	669	(40)	685	(43)	688	(40)
	Error rate	Same	12.3	(1.8)	18.6	(2.7)	12.4	(2.2)	17.7	(2.6)
		Different	13.9	(2.0)	10.8	(1.3)	11.7	(1.6)	11.4	(1.6)

The analysis of error rates did not yield any main effects of environment-related feature overlap, *F*(1,37) = 1.30, *p* = .26, η_p_^2^ = .03, body-related feature overlap, *F*(1,37) = 0.59, *p* = .45, η_p_^2^ = .02, or ISI, *F*(1,37) = 7.33, *p* = .01, η_p_^2^ = .17. However, as for RTs, the expected interaction between environment- and body-related feature overlap was significant, *F*(1,37) = 30.72, *p* < .001, η_p_^2^ = .45. Again, neither the interaction of ISI and environment-related feature overlap, *F*(1,37) = 0.86, *p* = .36, η_p_^2^ = .02, nor of ISI and body-related feature overlap, *F*(1,37) = 0.59, *p* = .45, η_p_^2^ = .02, nor the three-way interaction, *F*(1,37) = 2.78, *p* = .10, η_p_^2^ = .07, were significant.

#### Action A

As in Experiment 1, the RTs of action A (see Fig. 4, lower panel) were significantly lower when both actions had the same as opposed to a different environment-related effect, *F*(1,37) = 5.64, *p* = .02, η_p_^2^ = .13, and in trials with differing as opposed to identical body-related effects, *F*(1,37) = 41.72, *p* < .001, η_p_^2^ = .53. This, however, should be interpreted in the light of the significant cross-over interaction between environment- and body-related feature overlap, *F*(1,37) = 19.61, *p* < .001, η_p_^2^ = .35. The main effect of ISI was significant, *F*(1,37) = 4.86, *p* = .03, η_p_^2^ = .12, while the interactions between ISI and environment-related feature overlap, *F*(1,37) =.53, *p* = .47, η_p_^2^ = .01, ISI and body-related feature overlap, *F*(1,37) = 0.16, *p* = .70, η_p_^2^ < .01, and between all three factors were not, *F*(1,37) = 0.70, *p* = .41, η_p_^2^ = .02.

Also the analysis of error rates for action A yielded more accurate responses in environment-related effect repetition, *F*(1,37) = 5.67, *p* = .02, η_p_^2^ = .13, and finger-alternation trials, *F*(1,37) = 12.96, *p* = .001, η_p_^2^ = .26, as opposed to environment-related effect alternation and finger-repetition trials, respectively. Notably, also the expected interaction between these two factors was significant, *F*(1,37) = 23.12, *p* < .001, η_p_^2^ = .39. Further, there was no significant main effect of ISI, *F*(1,37) = 0.78, *p* = .38, η_p_^2^ = .02, no interaction between ISI and environment-related feature overlap, *F*(1,37) = 0.39, *p* = .54, η_p_^2^ = .01, or between ISI and body-related feature overlap, *F*(1,37) = 0.64, *p* = .43, η_p_^2^ = .02, and no three-way interaction between all factors, *F*(1,37) = 2.46, *p* = .13, η_p_^2^ = .06.

### Discussion

Experiment 2 replicated the results of Experiment 1. Again, the mean difference between action A and action B RTs was significant (see Table 4), showing that participants planned action A in advance.

Most importantly, initiation of action B was again delayed if it shared one as compared to both or neither feature of a concurrently planned action A, thereby indicating the temporary binding of body-related and environment-related features during action planning. Importantly, this was obtained while possible differences of varying display changes between the four crucial conditions were ruled out.

Regarding the main effects of environment- and body-related feature overlap, both were out significant for action A (surprisingly, with faster and more accurate responding in finger alternation than repetition trials). Importantly, for action B, features did not repeat or alternate with regard to a previously executed action (as was the case for action A), but with regard to a merely planned action. Remarkably, both main effects still seemed to have occurred for action B as well, although highly attenuated. To shed light on the functional equivalence benefit in action B, we combined the data of Experiments 1 and 2. Even this higher power analysis revealed only a tendency of faster initiation of action B if it aimed at the same environment-related effect as action A, *F*(1,63) = 2.97, *p* = .09, η_p_^2^ = .05.

Interestingly, while Stoet and Hommel (1999) observed a tendency towards partial overlap benefits for action A, our first two experiments yielded partial overlap costs similar to action B. Apparently, in the current design, action plans for action B were not yet disintegrated when participants were to perform action A.

## Experiment 3

Altogether, we found clear evidence for binding of environment-related action features during action planning when these features were equally task-relevant, that is, if they both had to be used for planning and retained for later action execution. This finding is, firstly, strong evidence against our body-relatedness hypothesis, as it is clearly possible for environment-related features to be bound to body-related features in the action planning process. Secondly, that a binding effect occurred in the present design with task-relevant body- and environment-related features but not in the study by Kunde et al. (2002) with a task-relevant body-related and a task-irrelevant environment-related feature is preliminary evidence for our task relevance hypothesis. However, the support for the task-relevance hypothesis would be even more convincing if rendering the environment-related features task-irrelevant removed indications of such binding (i.e., the characteristic interaction pattern of body-related and environment-related feature overlap). This is what we aimed to demonstrate in Experiment 3. Here we asked participants to plan an index or middle finger keypress for action A and to initiate such a keypress first for action B. Like in the previous experiments, the keypresses still produced towards or away movements of the cursor on the screen (depending on the stick figure position), but these movements were no more task-relevant.

### Method

#### Participants

To reach a sample size of *n* = 34 (corresponding to a power of 1–β = .80 of a two-sided paired-samples *t-*test to detect an effect of *d*_*z*_ = 0.50 with α = .05), it was necessary to recruit in total 46 participants (26 females, *M*_*age*_ = 28.0 years, *range*_*age*_ = 19–56 years). That is because we replaced 12 participants who failed to detect any catch-trials (i.e., those trials in which the task-relevant body-related feature differed between the first and last display).

#### Apparatus and stimuli

For this and the following experiment, stimuli were presented using the E-Prime 3.0 software (Psychology Software Tools, 2016). Moreover, while in Experiment 2 the cursor color indicated whether the cursor should move towards or away from the stick figure, in Experiment 3, the color indicated which finger should be used to initiate the cursor movement irrespective of the stick figure position. Specifically, the finger determined the movement direction, with an index (middle) finger keypress making the cursor move downwards (upwards) for action A and to the left (right) for action B. In a catch-trial, the cursor color in the final display suggested the use of another finger (i.e., body-related feature) than had been planned in the beginning. Unlike in the previous experiments, there were no more catch-trials in which the stick figure position and therefore the task-irrelevant environment-related feature (i.e., whether the cursor moved towards or away from the stick figure) changed. Besides that, the method was the same as in Experiment 2 (see Fig. 2b).

### Results

#### Action B

**Fig. 5:**
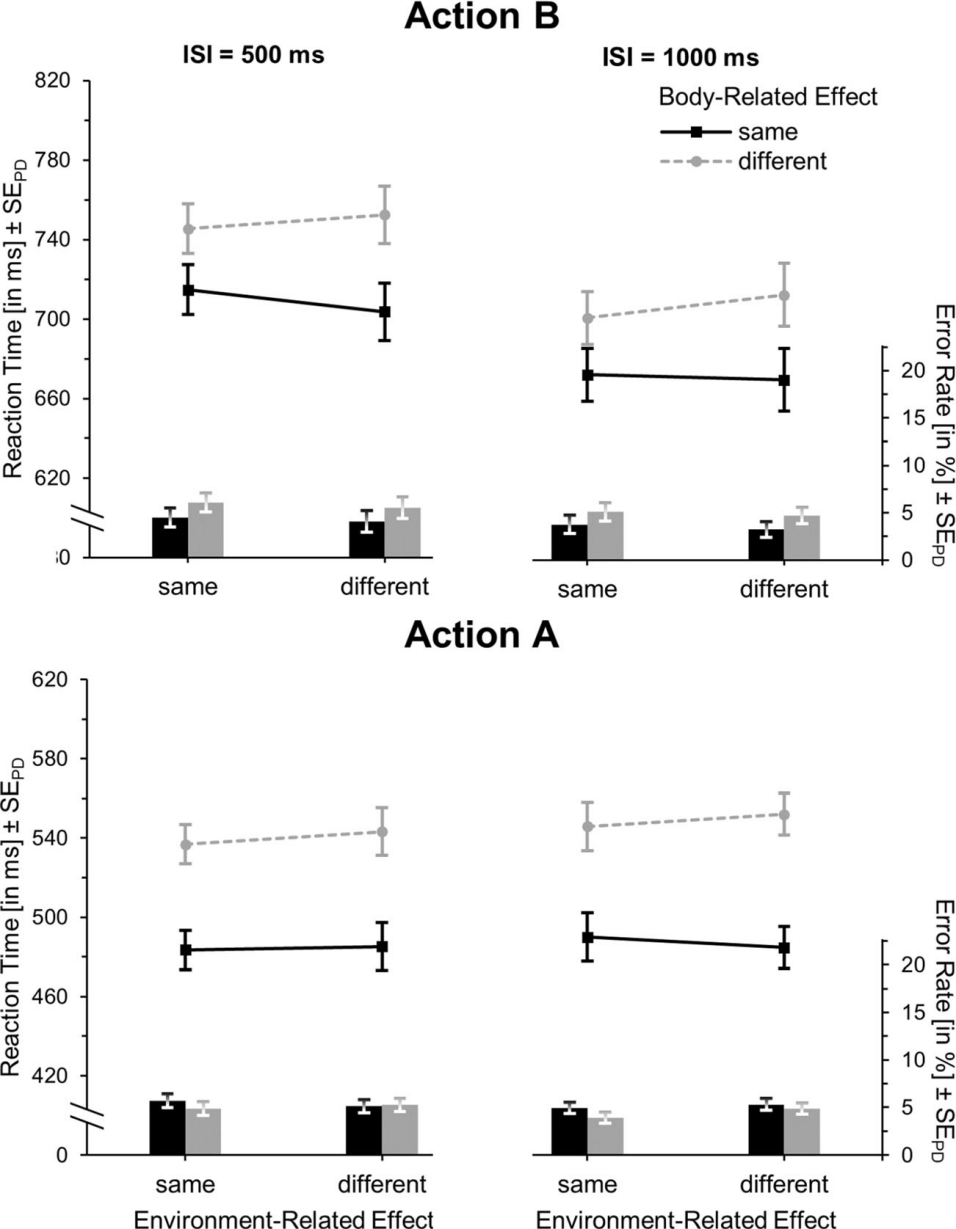
Mean reaction times (lines) and error rates (bars) for action B (upper panel) and action A (lower panel) in Experiment 3. Either both features (full repetition), no feature (full alternation) or only one of the features (partial overlap conditions) are identical in action A and action B. Within each environment-related feature-overlap condition, error bars represent standard errors of the paired differences (see Pfister & Janczyk, 2013) between the body-related feature-overlap conditions

The RT analysis of action B showed no main effect of environment-related feature overlap, *F*(1,33) = 0.09, *p* = .77, η_p_^2^ < .01, while responses were generally faster when the body-related effect repeated than when it differed, *F*(1,33) = 8.66, *p* < .01, η_p_^2^ = .21 (see Table 6 and Fig. 5, upper panel). Responding was overall faster with long rather than short ISI, *F*(1,33) = 36.37, *p* < .001, η_p_^2^ = .52. The crucial interaction pattern of body- and environment-related feature overlap of faster responding with full or no feature overlap as compared to partial overlap we found in Experiments 1 and 2 was removed, and in fact significantly reversed, *F*(1,33) = 6.06, *p* = .02, η_p_^2^ = .16. Particularly, responding was now faster with partial feature overlap (*M* = 706) as compared to full or no feature overlap (*M* = 714, *t*(33) = -2.46, *p* = .02, *d*_*z*_ = 0.42). Similar to the previous experiments, there were no significant interactions between ISI and environment-related feature overlap, *F*(1,33) = 0.85, *p* = .36, η_p_^2^ = .03, ISI and body-related feature overlap, *F*(1,33) = 0.29, *p* = .59, η_p_^2^ < .01, or between all three factors, *F*(1,33) = 0.09, *p* = .77, η_p_^2^ < .01.

**Table 6:** Means (and standard errors of the means) of reaction times (RTs) and error rates according to interstimulus interval (ISI), and environment-related and body-related feature overlap for actions A and B for Experiment 3 (n = 34)

			ISI							
			500 ms				1,000 ms			
			Environment-related effect				Environment-related effect			
		Body- related effect	Same		Different		Same		Different	
Action B	RT	Same	715	(17)	704	(19)	674	(17)	671	(17)
		Different	745	(20)	752	(22)	703	(20)	714	(22)
	Error rate	Same	4.2	(0.9)	3.8	(0.8)	3.8	(1.0)	3.2	(0.8)
		Different	5.8	(1.6)	5.2	(1.3)	5.1	(1.4)	4.7	(1.3)
Action A	RT	Same	483	(16)	485	(18)	490	(18)	485	(17)
		Different	537	(20)	543	(20)	546	(20)	552	(20)
	Error rate	Same	5.7	(0.8)	5.1	(0.7)	4.9	(0.7)	5.3	(0.8)
		Different	4.9	(0.8)	5.2	(0.8)	3.9	(0.5)	4.9	(1.0)

In error rates there were no main effects of environment-related feature overlap, *F*(1,33) = 1.77, *p* = .19, η_p_^2^ = .05, body-related feature overlap, *F*(1,33) = 2.83, *p* = .10, η_p_^2^ = .08, and ISI, *F*(1,33) = 3.96, *p* = .06, η_p_^2^ = .11. Also, there was neither an interaction of environment- and body-related feature overlap, *F*(1,33) = 0.01, *p* = .97, η_p_^2^ < .01, nor of ISI with environment-related, *F*(1,33) < 0.01, *p* = .99, η_p_^2^ < .01, or body-related feature overlap, *F*(1,33) = 0.03, *p* = .86, η_p_^2^ < .01, nor an interaction of all three factors, *F*(1,33) = 0.04, *p* = .84, η_p_^2^ < .01.

#### Action A

Action A was faster with repetition rather than alternation of the body-related action feature, *F*(1,33) = 31.88, *p* < .001, η_p_^2^ = .49, but no main effect of environment-related feature overlap, *F*(1,33) = 0.58, *p* = .45, η_p_^2^ = .02, and no interaction between environment- and body-related feature overlap occurred, *F*(1,33) = 2.49, *p* = .12, η_p_^2^ = .07 (see Fig. 5, lower panel). Responding was slightly faster with short as compared to long ISI, *F*(1,33) = 4.74, *p* = .04, η_p_^2^ = .13, while ISI interacted with neither environment-related feature repetition, *F*(1,33) = 0.37, *p* = .55, η_p_^2^ = .01, nor with body-related feature overlap, *F*(1,33) = 1.63, *p* = .21, η_p_^2^ = .05. There was also no three-way interaction, *F*(1,33) = 0.52, *p* = .48, η_p_^2^ = .02.

The analysis of error rates revealed no main effects of environment-related feature overlap, *F*(1,33) = 0.62, *p* = .44, η_p_^2^ = .02, body-related feature overlap, *F*(1,33) = 2.16, *p* = .15, η_p_^2^ = .06, or ISI, *F*(1,33) = 2.77, *p* = .11, η_p_^2^ = .08. Neither the interaction of environment- and body-related feature overlap was significant, *F*(1,33) = 2.10, *p* = .16, η_p_^2^ = .06, nor of ISI and environment-related, *F*(1,33) = 1.17, *p* = .29, η_p_^2^ = .03, or body-related feature overlap, *F*(1,33) = 0.37, *p* = .55, η_p_^2^ = .01, nor of all three factors, *F*(1,33) = 0.07, *p* = .79, η_p_^2^ < .01.

### Discussion

In Experiment 3, the environment-related feature of the actions (the towards or away movement displayed on the screen) was task-irrelevant. As a consequence, the characteristic pattern, that is, faster action B responses with full or no feature overlap with the concurrently planned action A as compared to partial feature overlap, was removed. It is certain that this is not due to a lack of planning, as also in this experiment participants actually planned action A in advance (see Table 4). We wanted to directly compare the obtained data patterns in Experiments 2 and 3. Notably, the designs only differed regarding the factor task relevance of the environment-related feature. Showing that the effects of partial feature overlap differ substantially between both would be strong evidence for our task relevance hypothesis. Indeed, the three-way interaction between Experiment (2 vs. 3), body-related feature overlap, and environment-related feature overlap was significant when entered into a post hoc between-experiment analysis, *F*(1,70) = 18.97, *p* < .001, η_p_^2^ = .21. Thus, these results support the idea that the environment-related features were no more bound to the body-related features during action planning. In fact, the crucial interaction pattern was now reversed, with faster responding in action B if there was partial as compared to no or full-feature overlap with action A.

The reasons for this significant reversal are not entirely clear at the moment. It might be that the repetition and change of the relevant body-related feature become mentally more distinct and hence affect performance more if the task-irrelevant environment-related feature changes rather than repeats. Whatever the reasons are, the reversal of this interaction as compared to Experiments 1 and 2 clearly suggests that the body-external feature was included in a different way in action planning, and not bound to the body-related feature. It is also noteworthy that we found a strong benefit of repeating the body-related feature in Experiment 3, which we did not observe in Experiments 1 and 2. What was apparent in Experiments 1 and 2 instead was that repetition of both task-relevant features (full repetitions) came with a benefit as compared to the combination of all other conditions. This suggests that the mental representation of the same efferent activity (i.e., a middle- or index-finger keypress) actually differed between Experiments 1 and 2 compared to Experiment 3. Generally, benefits occur if the action repeats, which was coded by a single feature in Experiment 3, but by a combination of features in Experiments 1 and 2.

## Experiment 4

We found no indication of binding of features of environment-related effects when these effects were task-irrelevant. Still, in the first two experiments, we found at least hints that action initiation tended to be faster when the to-be-initiated action B overlapped with the planned action A with regards to their environment-related feature. This raises the question whether preparing for a certain task-relevant environment-related action effect, without already binding it to a body-related feature, might increase such benefits or, to begin with, whether it is possible to prepare for a certain environment-related action effect without knowing which body movement will produce this effect. To address these questions, we asked participants to prepare a towards or away movement of the cursor but left unknown which finger will eventually be needed to produce that cursor movement by omitting the stick figure in the display for preparing action A. Thus, derived from TEC, an environment-related feature could be pre-activated but not yet bound to a body-related feature. There is already some evidence that participants can prepare certain abstract aspects of an action (such as the structure of elements of a motor sequence) without knowing the specific effectors to realize that action (Ulrich, Moore, & Osman, 1993; Ziessler, Hänel, & Sachse, 1990). The novel aspect here is that the prepared feature relates to a certain environment-related perceptual consequence of the movement, which, to our knowledge, has never been object of study before.

### Method

#### Participants

With the main effect of interest being the difference between the environment-related feature-overlap conditions in RTs for action B, a corresponding two-tailed matched-samples *t*-test would have a power of .80 to detect a medium-sized effect (i.e., *d*_*z*_ = 0.50 with α = .05) with *n* = 34 participants. We recruited 36 participants. While only one participant had less than 10 correct trials in at least one experimental cell, 14 other participants detected no catch-trials (i.e., those trials in which the environment-related feature changed from the beginning to the end of the trial). Hence, we excluded the participant with too many errors but, due to the unexpectedly high number of participants not detecting any catch-trials, we decided to retain those in the sample (*n* = 35, 29 females, *M*_*age*_ = 25.0 years, *range*_*age*_ = 19–52 years, 1–β = .82, α = .05, *d*_*z*_ = 0.50). While this strategy is rather conservative because it should decrease the size of our effect of interest (as participants who did not plan action A cannot be expected to show a functional equivalence benefit in action B), it allows for a closer look into the influence of the degree of planning on the effect of interest.

#### Apparatus and stimuli

As in Experiment 2, the external keys were aligned diagonally and the cursor color indicated the environment-related effect to be produced. However, as only the environment-related feature should be available when planning action A, display A initially contained the colored cursor, but not the stick figure (see Fig. 2c). Only at the end of each trial when participants executed the previously planned action A, the respective stick figure appeared in its position. Apart from this, the method and procedure were as in Experiment 2.

#### Data analysis

With the body-related effect not being available for planning action A, the factor body-related feature overlap was not included in the repeated-measures ANOVAs for action B. Still, it was part of the action A analyses. Cohen *d*_*z*_ will be additionally reported for the effect of main interest, that is, the functional equivalence benefit in RTs for action B, obtained by a post hoc two-tailed paired samples *t*-test.

### Results

#### Action B

**Fig. 6:**
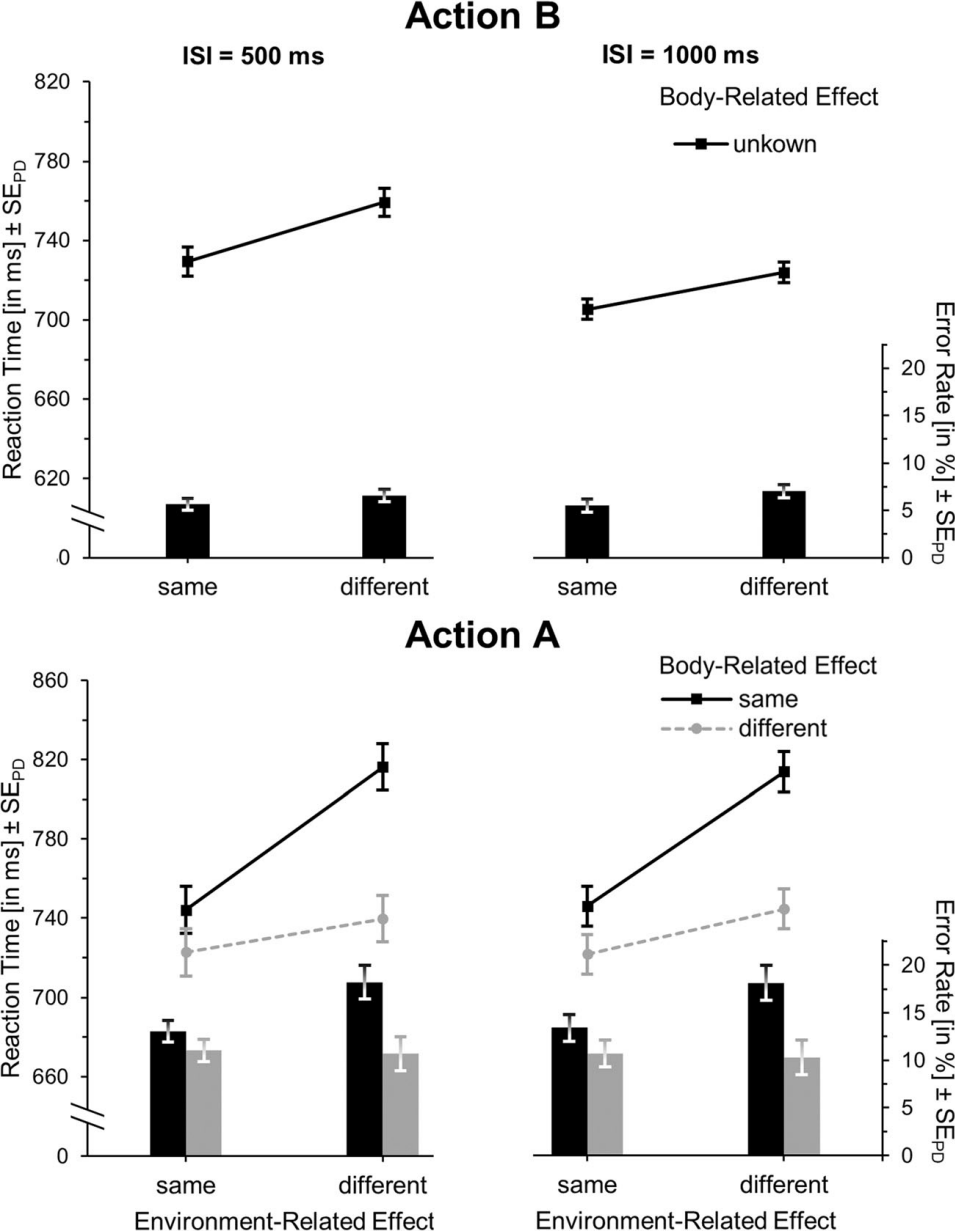
Mean reaction times (lines) and error rates (bars) for action B (upper panel) and action A (lower panel) in Experiment 4. In action A, either both features (full repetition), no feature (full alternation) or only one of the features (partial overlap conditions) are identical in action A and action B. Error bars represent standard errors of the paired differences (see Pfister & Janczyk, 2013) between the environment-related (action A) or body-related (action B) feature-overlap conditions, respectively

There was a significant main effect of environment-related feature overlap, *F*(1,34) = 21.21, *p* < .001, η_p_^2^ = .38, with lower RTs when action B aimed at the same (*M* = 718) rather than a different environment-related effect (*M* = 742, *t*(34) = -4.61, *p* <.001, *d*_*z*_ = 0.78) as action A (see Table 7 and Fig. 6, upper panel). Also, responses were faster with a long as compared to a short ISI, *F*(1,34) = 45.47, *p* < .001, η_p_^2^ = .57. These two factors did not significantly interact, *F*(1,34) = 2.80, *p* = .10, η_p_^2^ = .08. The analysis of error rates yielded the same main effect of environment-related feature overlap, *F*(1,34) = 6.84, *p* = .01, η_p_^2^ = .17, none of ISI, *F*(1,34) = 0.21, *p* = .65, η_p_^2^ < .01, and no interaction, *F*(1,34) = 0.43, *p* = .52, η_p_^2^ = .01.

**Table 7:** Means (and standard errors of the means) of reaction times (RTs) and error rates according to interstimulus interval (ISI), and environment-related and body-related feature overlap for actions A and B for Experiment 4 (n = 35)

			ISI								
			500 ms					1,000 ms			
			Environment-related effect					Environment-related effect			
		Body- related effect	Same		Different			Same		Different	
Action B	RT	Unknown	729	(21)	759	(23)		706	(20)	725	(21)
	Error rate	Unknown	5.6	(1.3)	6.5	(1.4)		5.5	(1.2)	7.0	(1.5)
Action A	RT	Same	744	(28)	816	(27)		746	(31)	814	(32)
		Different	723	(31)	740	(30)		722	(26)	744	(28)
	Error rate	Same	13.1	(1.8)	18.3	(2.6)		13.4	(2.0)	18.1	(2.6)
		Different	11.1	(1.6)	10.7	(1.3)		10.7	(1.5)	10.3	(1.4)

#### Action A

RTs of action A (see Fig. 6, lower panel) were lower when it aimed at the same rather than a different environment-related effect as action B, *F*(1,34) = 75.15, *p* < .001, η_p_^2^ = .69, and when it engaged a different as opposed to the same finger than action B, *F*(1,34) = 45.38, *p* < .001, η_p_^2^ = .57. This main effect of finger repetition was more pronounced when the environment-related effect differed than when it repeated, as shown in the significant interaction, *F*(1,34) = 16.24, *p* < .001, η_p_^2^ = .32. ISI produced neither a main effect, *F*(1,34) = 0.03, *p* = .86, η_p_^2^ < .01, nor interaction with the environment-related feature overlap, *F*(1,34) = 0.15, *p* = .90, η_p_^2^ < .01, nor body-related feature overlap, *F*(1,34) = 0.10, *p* = .75, η_p_^2^ < .01, nor a three-way interaction with these factors, *F*(1,34) = 0.32, *p* = .57, η_p_^2^ < .01.

Similarly, error rates were lower when action A aimed at the same as opposed to a different environment-related effect as action B, *F*(1,34) = 13.75, *p* = .001, η_p_^2^ = .29, and when it relied on a different rather than the same finger, *F*(1,34) = 18.21, *p* < .001, η_p_^2^ = .35, whereas these factors interacted in the same way as in the RT analysis, *F*(1,34) = 14.01, *p* = .001, η_p_^2^ = .29. There was no influence of ISI, *F*(1,34) = 0.11, *p* = .75, η_p_^2^ < .01, no interactions of ISI with environment-related feature overlap, *F*(1,34) = 0.12, *p* = .73, η_p_^2^ < .01, or with body-related feature overlap, *F*(1,34) = 0.42, *p* = .52, η_p_^2^ = .01, and no three-way interaction, *F*(1,34) = 0.02, *p* = .88, η_p_^2^ < .01.

#### Additional analysis

**Fig. 7:**
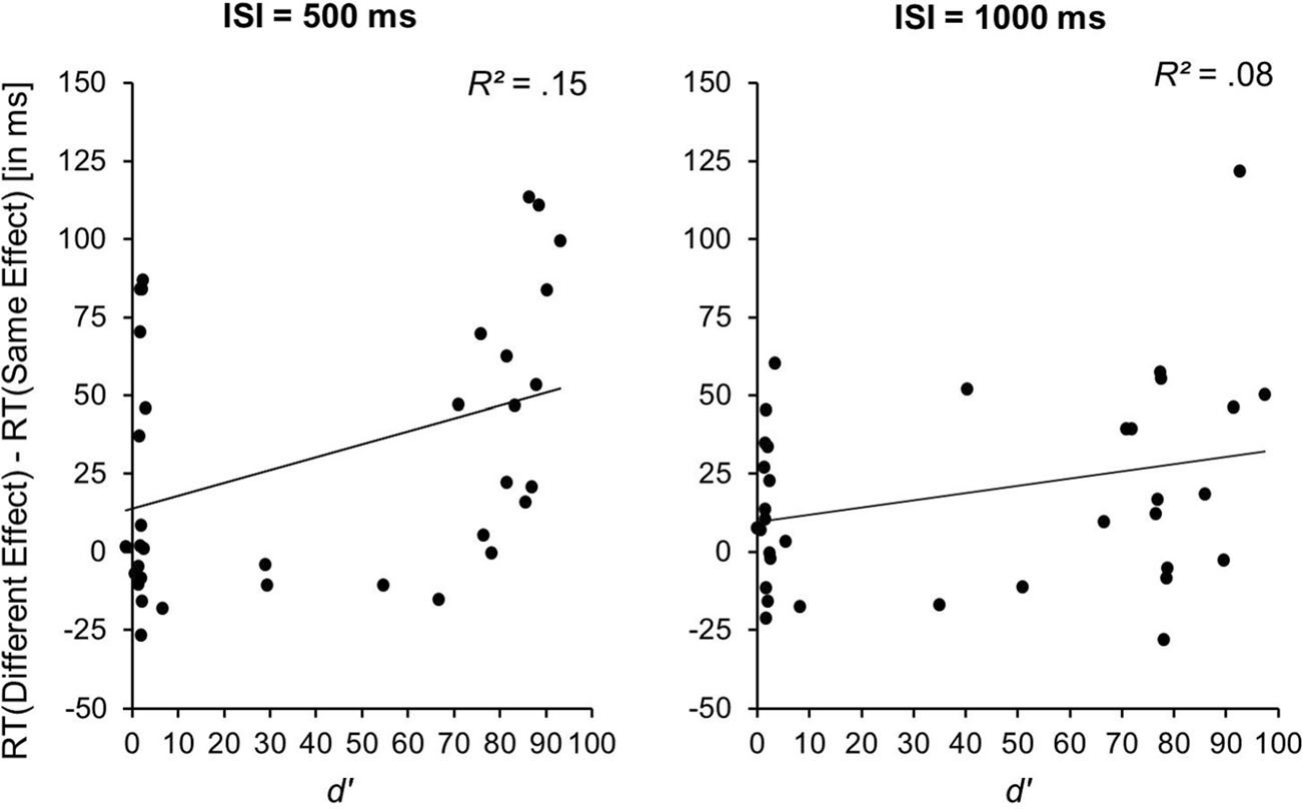
Individual functional equivalence benefit values reflected in reaction time differences between environment-related feature-overlap conditions by catch-trial detection performance as indicated by participants’ *d’* values

We conducted a post hoc analysis to reveal whether different degrees of planning (as indicated by the catch-trial detection performance in action A) influenced the size of the benefit of initiating an action B with the same rather than a different environment-related effect as action A (i.e., a functional equivalence benefit). Hence, for a short and long ISI we calculated the discrimination performance of catch-trials and non-catch-trials. To compute *d’* as indicator for catch-trial detection performance for each participant, we first excluded the practice blocks and RT outliers for action B. Then, we subtracted the false alarm rate (based on all non-catch-trials) from the hit rate (based on all catch-trials) and corrected values of 0 and 1 according to the log-linear rule (Goodman, 1970; Hautus, 1995). Across all participants, we then correlated *d’* in action A catch-trials, with the RT performance benefits in action B when initiating an action with the same as opposed to a different environment-related effect for both ISIs. As can be seen in Fig. 7, there were moderate positive correlations between these measures (in the long ISI condition, *r*(33) = .29, *p* = .10, and, significantly so, in the short ISI condition, *r*(33) = .38, *p* = .02^1^). Thus, the better the environment-related effect of action A was prepared, the larger was the benefit for initiating an action B that resulted in the same rather than a different environment-related effect.

### Discussion

Unlike in the previous experiments, the mean difference between action A and action B RTs did not yield a significant effect (see Table 4). This, however, is not too surprising, because only one feature of action A (as opposed to two features in the other experiments) could be prepared in advance, hence resulting in a smaller RT benefit. Experiment 4 revealed that, if it is task-relevant, an environment-related action effect feature can be pre-activated even if it is not possible to integrate it with a feature of a body-related action effect. Put differently, it yielded a strong benefit for initiating action B when concurrently preparing an action A that produced the same rather than a different environment-related effect, although the body-related means to produce that environment-related effect were yet unknown. This observation extends previous evidence showing that abstract aspects of a motor pattern can be prepared in advance, without knowledge of the muscles used to realize these motor patterns (Ulrich et al., 1993). The important point here is that this abstract feature related to a certain environment-related perceptual event. This supports the general idea that codes of perceptual events produced by efferent activities are involved in generating these efferent activities. Moreover, it suggests that functional equivalence benefits, that is, facilitated initiation of actions that produce a similar rather than a dissimilar environment-related effect as a concurrently planned action (observed as a tendency in Experiments 1 and 2), increase when feature codes of these environment-related effects are task-relevant but not yet bound to body-related features. We will discuss this topic further in the *General discussion* section.

## General discussion

The present research derived from the assumption that actions are planned by temporarily binding features of to be-produced perceptual effects. More precisely, taking up the ideo-motor idea, we presumed that a motor response can only be mentally prepared by anticipating the perceptual experience associated with the movement. Thus, planning for example a left index finger movement is not based on neural codes or muscular innervation patterns. Instead, such action planning relies on anticipations of body-related action effects (e.g., the change in the respective finger’s visual appearance or the proprioceptive or tactile experience associated with the movement) and, as shown, potentially also anticipations of environment-related effects such as a cursor movement on screen.

Specifically, we studied the role of task relevance and body-relatedness of such action effects for inclusion of their respective features in action plans. The results very consistently revealed that features denoting body- and environment-related effects become bound into action plans, providing they are task-relevant (Experiments 1 and 2). The present experiments are the first to reveal this by a full design including both full and partial repetitions as well as full alternations of effect features while previous research had relied on limited comparisons of full and partial feature alternations alone.

If task-relevant, including a certain environment-related feature facilitates functionally equivalent motor patterns, that is, actions that aim at producing the same environment-related event, provided that environment-related feature is not already bound to another feature (Experiment 4). By contrast, environment-related features that are task-irrelevant are apparently neither bound to action plans nor do they facilitate motor activities that rely on the same feature (Experiment 3).

Notably, despite differences in RTs, all these observations were obtained already 1,500 ms after the prepared event had been announced, and did not change dramatically with a longer delay of 2,000 ms, suggesting that the respective action planning processes had already matured after 1,500 ms. As our ISI manipulation did not significantly affect our effects of interest, future investigations should select better spaced intervals in order to get a deeper insight into the time course of the planning process. Shorter intervals for instance might result in pre-activation but not yet binding of action effect features as observed by Stoet and Hommel (1999).

It should be further mentioned that participants possibly categorized colors according to their meaning. In Experiments 1, 2, and 4, the color category signaled the required environment-related effect (i.e., towards or away). This might partially explain differences between environment-related feature repetition and alternation conditions, which seemed to be more pronounced for action A (i.e., when the final color category had already occurred twice in the same trial as opposed to only once for action B). The same mechanism might have played a role in Experiment 3, in which the category indicated the required body-related effect (i.e., index or middle finger).

One peculiarity of those body-related action effects that the present and also previous research have been focusing on is that the body-related sensory feedback on which action plans were based arose directly from the motor response. More specifically, the (e.g., visual, proprioceptive or tactile) sensation of a, for instance, left index finger movement resulted from pressing a key with this exact finger. Certainly, feeling the consequences of a movement directly on the effector that generated this movement should be the most natural form of body-related action effects. Still, Pfister, Janczyk, Gressmann, Fournier, and Kunde (2014) showed that, similar to environment-related action effects, vibrotactile (i.e., body-related) effects at one effector can shape the production of motor responses generated by a different effector. This is well in line with our finding that body-related and environment-related action effects have similar potential for binding in action plans.

Altogether, it is therefore task relevance rather than body-relatedness of a feature that determines whether or not this feature is bound to an action plan. Under appropriate conditions, benefits of generating an action that aims at the same environment-related effect as a planned action can be obtained as well. This data pattern thus reconciles seemingly contradictory previous results. Features of environment-related action effects become part of action plans (as suggested by Stoet & Hommel, 1999) while they can also facilitate functionally equivalent actions, that is, environment-related effects that comprise of the same features (as suggested by Kunde et al., 2002). That task relevance drives the binding effects here fits well to other, related binding effects in different action control tasks. For instance, Hommel (2005) argued that task relevance in terms of intentional weighting determines local bindings of stimulus features and of response features (see Memelink & Hommel, 2013, for a discussion). In the same vein, Singh and colleagues argued that irrelevant stimulus features only become bound (and thereby modulate responding later on) if they were attended (Singh, Moeller, Koch, & Frings, 2018) in the distractor-response-binding task (e.g., Frings et al., 2007).

Against this background of converging evidence of task relevance in binding tasks, the present results contribute to a general framework of action control, namely Binding and Retrieval in Action Control (BRAC), we have suggested recently (Frings, Hommel, et al., 2020a; Frings, Koch, et al., 2020b). This framework holds that many phenomena in which repeated actions are required entail two key processes, namely binding of features in a previous S-R-E episode, and retrieval of all features of that episode if at least one of them repeats in a current episode. This retrieval of previously used features creates problems if they are not needed in the current episode. Planning an action (A) can be construed as such an episode, though one that has not yet occurred. In this respect, the present observations corroborate recent evidence that, besides in perceiving, feature binding is also involved in imagining corresponding events (Cochrane & Milliken, 2019). Initiating another action (B) retrieves that mental episode if some features overlap, invoking costs of partial feature overlap because the to-be-initiated action reactivates other, not yet required features of the planned action. This interpretation is slightly different, though not at all incompatible, to the idea that features of planned actions are less accessible to actions that require these features as well. Still, it makes sense to conceptually tell apart these two processes as feature occupation on the one hand and involuntary feature retrieval on the other hand.

One implication of this distinction for the interpretation of Experiment 3 is as follows: If involuntary feature retrieval was the mechanism underlying partial overlap costs rather than feature occupation, it might be that irrelevant features (e.g., a towards or away movement) are still bound to relevant features (e.g., a finger movement) while planning action A, but that initiating action B fails to retrieve the irrelevant features of action A. In short, task relevance of features would not shape the binding process itself but rather determine subsequent retrieval of these features from the formed action plan. Future research is warranted to set apart these processes not only conceptually but experimentally.

Furthermore, future research should attempt to identify moderators of binding and/or retrieval. For instance, one might assume that these processes could be facilitated when affectively enriching action A, for instance by assigning a positive or negative valence to the cursor and an approach or avoidance meaning to the environment-related effect. It should be further noted that an important limitation of the present paradigm is that, similarly to most previous investigations (e.g., Fournier et al., 2015; Kunde et al., 2002; Stoet & Hommel, 1999), it merely applied spatial and quasi-spatial features. Thus, it should be worthwhile to systematically study whether the nature of action effects influences binding processes for example by means of temporal, auditory or valence feature dimensions.

### Conclusion

The present research showed that an action plan can not only comprise bound features of body-related, but also environment-related action effects. Importantly, for this binding (or at least retrieval of such binding in subsequent action planning) to occur, these features must be relevant for the task at hand. In addition to these main findings, performing an action can benefit if it aims at the same rather than a different environmental effect as a previously planned one. Overall, while the presented experiments brought seemingly contradictory findings into accordance, they certainly raised new questions that future research will need to address.

#### Authors Note

This research was supported by the German Research Council (DFG) within the research unit FOR 2790 Binding and Retrieval in Action Control (grant KU 1964/ 17-1).

#### Open Practices Statement

The data and syntaxes for statistical analyses of all experiments, as well as the preregistrations for Experiments 3 and 4 are publicly available on the Open Science Framework (https://osf.io/3xush/).

## Footnotes

1 Excluding those participants who detected no catch trials at all leads to even higher correlations (in the long ISI condition, *r*(19) = .35, *p* = .12, and, significantly so, in the short ISI condition, *r*(19) = .44, *p* < .05).
